# Gain-of-function mutations in *KCNK3* cause a developmental disorder with sleep apnea

**DOI:** 10.1038/s41588-022-01185-x

**Published:** 2022-10-04

**Authors:** Janina Sörmann, Marcus Schewe, Peter Proks, Thibault Jouen-Tachoire, Shanlin Rao, Elena B. Riel, Katherine E. Agre, Amber Begtrup, John Dean, Maria Descartes, Jan Fischer, Alice Gardham, Carrie Lahner, Paul R. Mark, Srikanth Muppidi, Pavel N. Pichurin, Joseph Porrmann, Jens Schallner, Kirstin Smith, Volker Straub, Pradeep Vasudevan, Rebecca Willaert, Elisabeth P. Carpenter, Karin E. J. Rödström, Michael G. Hahn, Thomas Müller, Thomas Baukrowitz, Matthew E. Hurles, Caroline F. Wright, Stephen J. Tucker

**Affiliations:** 1 Clarendon Laboratory, Department of Physics, University of Oxford, Oxford, UK; 2 Kavli Institute for Nanoscience Discovery, University of Oxford, Oxford, UK; 3 Institute of Physiology, Faculty of Medicine, Kiel University, Kiel, Germany; 4 Department of Pharmacology, University of Oxford, Oxford, UK; 5 OXION Initiative in Ion Channels and Disease, University of Oxford, Oxford, UK; 6 Department of Biochemistry, University of Oxford, Oxford, UK; 7 Mayo Clinic, Rochester, MN USA; 8 GeneDx, Gaithersburg, MD USA; 9 Department of Medical Genetics, NHS Grampian, Aberdeen, UK; 10 Department of Genetics, University of Alabama at Birmingham, Birmingham, AL USA; 11 Institute for Clinical Genetics, Universitätsklinikum, Technischen Universität Dresden, Dresden, Germany; 12 North West Thames Regional Genetics Service, London North West Healthcare NHS Trust, London, UK; 13 Spectrum Health Medical Genetics, Grand Rapids, MI USA; 14 Stanford Neurosciences Health Center, Palo Alto, CA USA; 15 Department of Neuropediatrics, Universitätsklinikum, Technischen Universität Dresden, Dresden, Germany; 16 Institute of Translational and Clinical Research, Newcastle University, Newcastle upon Tyne, UK; 17 University Hospitals of Leicester NHS Trust, Leicester Royal Infirmary, Leicester, UK; 18 Centre for Medicines Discovery, University of Oxford, Oxford, UK; 19 Bayer AG, Research & Development, Pharmaceuticals, Wuppertal, Germany; 20 Human Genetics Programme, Wellcome Sanger Institute, Wellcome Genome Campus, Cambridge, UK; 21 Institute of Biomedical and Clinical Science, University of Exeter Medical School, Exeter, UK

**Keywords:** Biophysics, Genetics, Diseases, Biochemistry, Physiology

## Abstract

Sleep apnea is a common disorder that represents a global public health burden. *KCNK3* encodes TASK-1, a K^+^ channel implicated in the control of breathing, but its link with sleep apnea remains poorly understood. Here we describe a new developmental disorder with associated sleep apnea (developmental delay with sleep apnea, or DDSA) caused by rare de novo gain-of-function mutations in *KCNK3*. The mutations cluster around the ‘X-gate’, a gating motif that controls channel opening, and produce overactive channels that no longer respond to inhibition by G-protein-coupled receptor pathways. However, despite their defective X-gating, these mutant channels can still be inhibited by a range of known TASK channel inhibitors. These results not only highlight an important new role for TASK-1 K^+^ channels and their link with sleep apnea but also identify possible therapeutic strategies.

## Abstract (web summary)

Heterozygous de novo gain-of-function mutations in *KCNK3*, which encodes the two-pore-domain K^+^ channel TASK-1, cause a channelopathy characterized by developmental delay with sleep apnea.

## Main

Sleep apnea is thought to affect up to 1 billion people worldwide and is characterized by abnormal, interrupted breathing during sleep^1,2^. The poor quality of sleep that arises results in a huge economic and societal impact and a decreased quality of life and increases the risk of comorbidities, such as cardiovascular disease, diabetes and depression, as well as the risk of motor vehicle accidents^3^. Sleep apnea is therefore a major public health burden, and there is a large unmet clinical need for more effective treatments^4^. However, it is also a complex disorder, and the underlying mechanisms are often unclear.

Increasing evidence suggests that instability of ventilatory control is involved in the pathogenesis of both central and obstructive sleep apnea, the two principal forms of the disorder^2^. Peripheral and central chemoreceptors, which detect O_2_/CO_2_ levels, also have a critical role, although their molecular identity and the neuronal networks involved remain poorly defined^5^. Likewise, defective muscle control of the nasopharynx can also contribute to collapse of the upper airway and obstructive sleep apnea. Thus, defects in respiratory drive and/or nasopharyngeal muscle tone can be underlying causes of sleep apnea, and in some cases, a complex phenotype with features of both central and obstructive sleep apnea is reported^6^.

Two-pore-domain K^+^ (K2P) channels are a structurally distinct subset of K^+^ channels where each gene encodes a subunit with two pore-forming domains that co-assemble as a ‘dimer of dimers’ to create a single pseudotetrameric K^+^-selective channel across the membrane^7,8^. K2P channels underlie the background K^+^ currents that control the membrane potential in many different cell types. Originally described as ‘leak’ channels, their activity is now known to be regulated by diverse stimuli, including many G-protein-coupled receptor (GPCR) pathways, thus enabling them to integrate different neuronal, metabolic and cellular signaling pathways into changes in cellular electrical activity^9,10^. Such regulatory pathways are important in many of the neural systems that regulate breathing^11^, including the control of respiratory drive immediately after birth, where dysfunctional regulation of this pathway gives rise to an increased frequency of spontaneous apneas^12^.

In particular, *KCNK3* encodes the TASK-1 K2P channel and is expressed in a variety of neuronal populations throughout the central nervous system (CNS), including in many chemosensitive regions involved in the regulation of ventilation, as well as in hypoglossal and spinal cord motor neurons^13–15^. In peripheral tissues, TASK-1 is also found in the carotid bodies, lung, heart and pulmonary arterial smooth muscle^16–18^. Its expression throughout cells/tissues involved in both the control of respiratory drive and mechanical ventilation has therefore implicated TASK-1 in sleep apnea^13,19–23^. Furthermore, an X-ray crystal structure of the TASK-1 channel has recently been reported in complex with a compound class of TASK-1 inhibitors used in clinical trials for the treatment of sleep apnea^24^. This structure also revealed several unique features of TASK-1, including a lower ‘X-gate’, a structural motif that controls opening and closing of the channel pore^24^.

However, a clear mechanistic link between TASK-1 and sleep apnea remains unproven and is further complicated by the propensity of TASK-1 subunits to co-assemble with related TASK-3 (*KCNK9*) subunits to form new heteromeric TASK-1–TASK-3 channels in cells where both genes are coexpressed^25,26^. Moreover, heterozygous loss-of-function variants in *KCNK3* are associated with a different disorder, pulmonary arterial hypertension (PAH), an adult-onset, progressive and often fatal disease characterized by increased pulmonary arterial pressure in the absence of the common causes of pulmonary hypertension^27^; in addition, loss-of-function variants in the TASK-3 channel (*KCNK9*) are associated with a neurodevelopmental disorder, Birk-Barel syndrome^28^.

In this study, we describe nine probands with de novo missense mutations in *KCNK3* who exhibit global developmental delay, hypotonia, a range of structural malformations and sleep apnea. The mutations all cluster near the recently identified lower X-gate of the TASK-1 channel and result in a new gain-of-function phenotype that appears to correlate with the severity of the disorder. These results have important implications for the treatment of these probands and other individuals with sleep apnea, as well as for our understanding of the role that TASK-1 channels have in cellular function.

## Results

### De novo mutations in *KCNK3* cause developmental delay with sleep apnea

**Fig. 1:**
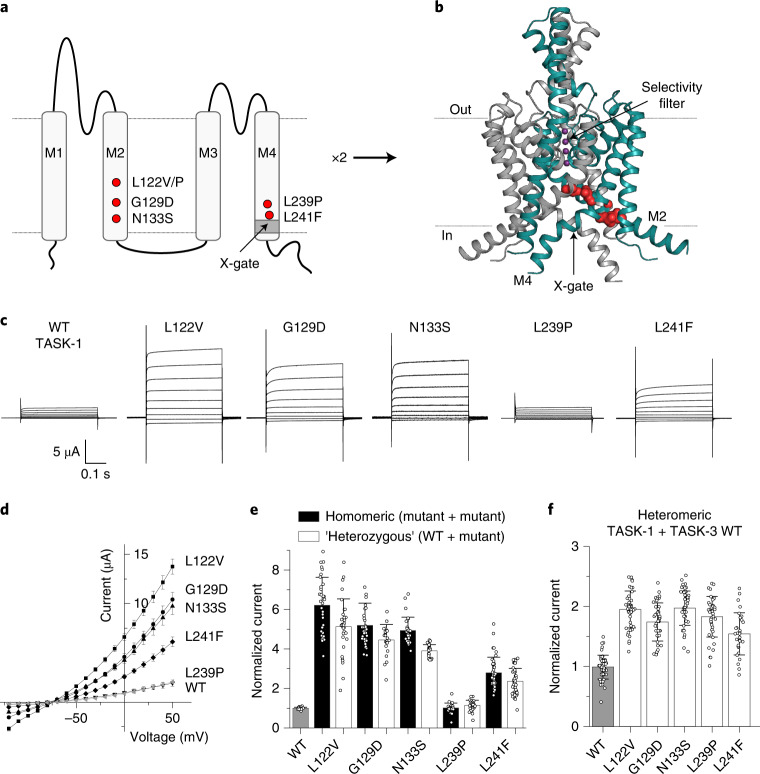
DDSA mutations produce a gain-of-function phenotype in TASK-1. **a**, Topological model of a TASK-1 subunit with the position of the DDSA variants labeled in red and the X-gate in dark gray. Two subunits co-assemble to form the K^+^ channel pore. **b**, Model showing the dimeric structure of TASK-1 (PDB: 6RV2), with one subunit shown in teal and the residues mutated in DDSA shown as red spheres. K^+^ ions within the selectivity filter are shown in purple. **c**, Representative TEVC recordings of WT TASK-1 and DDSA mutant currents in response to voltage steps from −120 to +50 mV in 20 mV steps from a holding potential of −80 mV. **d**, Current–voltage plot of WT TASK-1 (*n* = 38), L122V (*n* = 39), G129D (*n* = 44), N133S (*n* = 55), L241F (*n* = 42) and L239P (*n* = 57); data are presented as mean ± s.d. **e**, Currents for homomeric DDSA mutants, and ‘heterozygous’ channels formed from 1:1 coexpression of WT TASK-1 and DDSA mutants normalized to WT current at +50 mV: WT (*n* = 28), L122V (*n* = 32), L122V–WT (*n* = 32), G129D (*n* = 36), G129D–WT (*n* = 23), N133S (*n* = 29), N133S–WT (*n* = 23), L239P (*n* = 25), L239P–WT (*n* = 34), L241F (*n* = 42) and L241F–WT (*n* = 43), data are presented as mean ± s.d. With the exception of L239P, all mutant currents differ from WT (*P* < 0.01, two-paired *t*-test). **f**, WT or DDSA mutant TASK-1 coexpressed 1:1 with WT TASK-3. Currents normalized to WT heteromeric TASK-1–TASK-3 currents: WT (*n* = 50), L122V (*n* = 49), G129D (*n* = 44), N133S (*n* = 53), L239P (*n* = 41) and L241F (*n* = 30), data are presented as mean ± s.d. All mutant currents differ from WT (*P* < 0.01, two-paired *t*-test).

A recent analysis of 31,058 parent–offspring trios with severe developmental disorders identified 28 new disease-causing genes with a high burden of de novo mutations, including a disorder caused by recurrent missense variants in *KCNK3* (ENST00000302909; NM_002246)^29^. Through parent–offspring exome sequencing performed across four different diagnostic laboratories and research studies, we have now identified a total of nine probands, each heterozygous for one of six de novo missense variants in *KCNK3* (Methods and Table 1). These new variants were found to cluster in two regions of the protein (ENSP00000306275.3; NP_002237): L122V, L122P, G129D (two probands) and N133S (three probands) are in the second transmembrane helix (M2), while L239P and L241F are in the fourth transmembrane helix (M4) (Fig. 1a,b).

**Table 1:** Summary of *KCNK3* variants and phenotypes in nine probands identified with DDSA

Characteristic	Proband 1	Proband 2	Proband 3	Proband 4	Proband 5	Proband 6	Proband 7	Proband 8	Proband 9
Position (GRCh37)	Chr2:26950615	Chr2:26950616	Chr2:26950637	Chr2:26950637	Chr2:26950649	Chr2:26950649	Chr2:26950649	Chr2:26950967	Chr2:26950972
HGVSc (NM_002246; ENST00000302909)	c.364C>G	c.365T>C	c.386G>A	c.386G>A	c.398A>G	c.398A>G	c.398A>G	c.716T>C	c.721C>T
HGVSp (NP_002237; ENSP00000306275)	p.Leu122Val (**L122V**)	p.Leu122Pro (**L122P**)	p.Gly129Asp (**G129D**)	p.Gly129Asp (**G129D**)	p.Asn133Ser (**N133S**)	p.Asn133Ser (**N133S**)	p.Asn133Ser (**N133S**)	p.Leu239Pro (**L239P**)	p.Leu241Phe (**L241F**)
Inheritance	De novo	De novo	De novo	De novo	De novo	De novo	De novo	De novo	De novo
Proband sex	Male	Male	Male	Male	Male	Male	Female	Female	Female
Age	18 years	3 years	(21 weeks)	9 years	10 years	9 years	10 years	5 years	25 years
Sleep apnea*	Central	Central	NA	Central	Central	Central	Central/ obstructive	Central/ obstructive	Obstructive
AHI*	37	54	NA	(Not measured)	11	21	31	6	2
Nadir SpO_2_*	80%	67%	NA	(Not measured)	75%	82%	71%	85%	92%
Nocturnal O_2_	Yes	Yes	NA	PEEP	Yes	CPAP	BiPAP	Yes	BiPAP
Developmental delay	Mild	Severe	NA	Severe	Moderate	Moderate	Mild	Mild	Mild
Learning difficulties	ASD, learning difficulties, speech delay	Speech and language delay, absent speech	NA	Absent speech	ADHD, learning difficulties, speech delay	–	Speech and language delay	Speech and language delay	Speech and language delay, mildly dysarthric
Abnormality of musculoskeletal system	Hypotonia, fatiguability, mild scoliosis, torticollis	Hypotonia	Flexion contractures, axillary and perineal pterygia	Hypotonia, arthrogryposis with joint pterygia, severe scoliosis	Hypotonia	Hypotonia, joint hypermobility	Hypotonia, fatigability	Hypotonia	Hypotonia
Abnormality of limbs	Talipes calcaneovalgus	Bilateral foot deformities, progressive cavovarus	Rocker bottom foot	Bilateral talipes equinovarus	Bilateral talipes equinovarus. Left foot fixed, right positional.	Bilateral talipes	Bilateral talipes	–	-
Abnormality of male genitalia/groin	–	Bilateral undescended testes	Ambiguous genitalia, penile hypospadias	Undescended testes, scrotal hypoplasia	Undescended testes (corrected)	Undescended testes (corrected)	NA	NA	NA
Abnormality of digestive system	Diarrhea, constipation, poor feeder	Constipation	NA	Dysphagia, gastroesophageal reflux	Constipation, feeding difficulties	–	Constipation	Constipation, dysphagia	Constipation, dysphagia
Abnormality of face/jaw	Facial asymmetry, High arched palate	Wide-set eyes, upturned nose, thin upper lip, myopathic facies	Hypertelorism, Micrognathia, Cleft palate, Anteverted nares	Bilateral cleft palate	Deeply set eyes, wide mouth	Tented upper lip vermilion, myopathic facies	Facial weakness, hypoplastic tooth enamel	Facial weakness, tented upper lip, high arched brows	Drooping of both eyelids, long narrow face
Pregnancy and birth	–	IUGR, NICU (85 days) polyhydramnios	IUGR, termination of pregnancy	C-section, IUGR, polyhydramnios	–	–	C-section, polyhydramnios	C-section, NICU (8 days)	C-section, NICU (7 days)
Weight (current)	−1.79 s.d.	+1.37 s.d.	<−2 s.d.	−3.68 s.d.	−0.25 s.d.	−0.63 s.d.	−0.95 s.d.	+0.25 s.d.	−5.07 s.d.
Height (current)	−2.27 s.d.	+0.37 s.d.	NA	−2.28 s.d.	+0.42 s.d.	Unknown	+0.58 s.d.	−0.35 s.d.	+0.20 s.d.
OFC (current)	−2.20 s.d.	No recent	<−2 s.d.	−3.08 s.d.	−1.55 s.d.	−1.07 s.d.	−0.61 s.d.	−0.10 s.d.	No recent

*Original full sleep reports for 3/8 living probands are reproduced in Supplementary Data 1. For the remaining probands, only AHI and nadir SpO_2_ values remain available after their original sleep apnea diagnoses. All AHI and SpO_2_ values were measured before any treatment. **Acronyms**: AHI, apnea-hypopnea index; SpO_2_, oxygen saturation; PEEP, positive end-expiratory pressure; CPAP, continuous positive airway pressure; BiPAP, bilevel positive airway pressure; OFC, occipital frontal circumference; NICU, neonatal intensive care unit; ASD, autism spectrum disorder; ADHD, attention deficit hyperactivity disorder; C-section, cesarean section; IUGR, intrauterine growth restriction.

All nine probands share similar phenotypes that define this new rare monogenic channelopathy, which we henceforth refer to as ‘developmental delay with sleep apnea’ (DDSA) (Table 1). All living probands (8/9, aged 3–25 years) had hypotonia, global developmental delay, central and/or obstructive sleep apnea, and feeding difficulties that necessitated the insertion of a percutaneous endoscopic gastrostomy tube. One proband was terminated at 21 weeks due to abnormalities detected during routine ultrasound scanning. The majority of probands (7/9) had major structural malformations, including microcephaly, arthrogryposis/flexion contractures, scoliosis, cleft palate and bilateral talipes, with some facial dysmorphology, and the male probands (5/6) had ambiguous genitalia. Two probands (aged 5 and 25 years) are notably less severely affected, with feeding difficulties, hypotonia and sleep apnea, but only mild developmental delay and no structural abnormalities. Intriguingly, these two phenotypically milder mutations (L239P, L241F) are located in the M4 helix of the TASK-1 protein, whereas those in the more severely affected probands (L122V/P, G129D and N133S) are all located in the M2 helix. Two of these probands, with a recurrent M2 mutation (G129D), also appear to be the most severely affected with additional arthrogryposis and pterygia.

All eight living DDSA probands were diagnosed with sleep apnea following evaluation in a clinical sleep laboratory. Apnea-hypopnea index (AHI) values and oxygen saturation nadirs are shown for all probands (Table 1), and examples of polysomnography reports obtained before treatment are shown in Supplementary Data 1. Although other developmental disorders have been linked with sleep disturbances, the universality of sleep apnea in 100% of probands with pathogenic *KCNK3* mutations is unusual. Within the United Kingdom-based Deciphering Developmental Disorders (DDD) cohort of >13,500 probands with severe developmental disorders, just 0.6% of probands have sleep apnea recorded (0.06% with central sleep apnea). Furthermore, no causal gene (except for *KCNK3*) has sleep apnea recorded in every DDD proband with a diagnostic variant.

Interestingly, in keeping with the observed genotype–phenotype correlation for M2 versus M4 variants, the two probands with variants in the M4 helix were only mildly affected with central and/or obstructive sleep apnea (AHI values, 2–6), while the other six probands with variants in the M2 helix are severely affected with central sleep apnea (AHI values, 11–52), requiring ongoing treatment with nocturnal O_2_ through bilevel or continuous positive air pressure (BiPAP or CPAP) or pressure-controlled ventilation in the most severe case.

### DDSA variants cluster around the X-gate in TASK-1

Most K2P channels are thought to open and close by controlling a ‘gate’ within their K^+^ selectivity filter^30–32^. However, a recent structure of TASK-1 revealed a new lower ‘X-gate’ formed by an interaction of the pore-lining M4 helices that occludes the entrance to the pore^24^. This is also consistent with several reports that filter gating may not be the dominant form of gating in TASK channels^33,34^. Notably, this closed X-gate structure contains a motif (VLRFMT) previously implicated in the control of channel activity by volatile anesthetics and GPCRs, although the mechanisms involved remain unclear^35,36^.

On examination of the TASK-1 crystal structure (Protein Data Bank (PDB): 6RV2), we found that, even although the mutated residues are on two different transmembrane helices (M2 and M4), they all cluster near the X-gate and are involved in inter- and/or intra-subunit interactions, likely to hold the X-gate closed. Mutation affecting these residues is therefore likely to disrupt the closed state structure and increase the frequency of channel opening (Fig. 1b and Supplementary Fig. 1). Given that several of the de novo mutations are recurrent and that loss-of-function variants in *KCNK3* are associated with a completely different adult-onset disorder, PAH^27^, we hypothesized that DDSA may be caused by a different functional defect.

### DDSA mutations produce a gain-of-function phenotype in TASK-1

To examine this hypothesis, we measured the functional activity of these DDSA mutant channels by heterologous expression of either wild type (WT) or mutant TASK-1 channels in *Xenopus* oocytes. Two-electrode voltage clamp (TEVC) recordings revealed whole-cell K^+^ currents markedly larger than those with WT TASK-1. Only one variant, L239P in the M4 helix, produced whole-cell currents similar in size to those of WT TASK-1 (Fig. 1c,d and Supplementary Fig. 2a).

The mutant channels all exhibited reversal potentials around −80 mV in low external K^+^ concentrations, consistent with K^+^-selective channels (Fig. 1d). The L122V variant produced the largest increase in current compared to WT, consistent with the activatory effect of variants at the structurally equivalent position in many other K2P channels^37^. Furthermore, in agreement with the observed differences in disease severity for the M2 versus M4 variants, those in M2 produced the largest increase in current compared to WT TASK-1 (3.2- to 6.2-fold), whereas currents for the two M4 variants were either similar in size to those for WT (that is, L239P) or only ~2.8-fold increased (that is, L241F; Fig. 1d).

### Gain of function in homomeric, ‘heterozygous’ and heteromeric TASK channels

K2P channel subunits assemble as dimers, and the results described above were all obtained in homomeric channels, where both subunits contained the variant. However, the DDSA probands are heterozygous for a single mutation, meaning that a mixture of channels can be created from co-assembly of WT and mutant subunits (25% WT, 50% ‘heterozygous’ and 25% ‘homomeric’ mutant). Therefore, to better replicate this heterozygous genotype, we co-injected oocytes with WT TASK-1 mRNA plus an equal amount of either WT or mutant mRNA in a 1:1 ratio. In all cases, the resulting currents were at least 80% the size of the currents formed by the homomeric mutant channels (Fig. 1e). An increase in current was also observed for the most common DDSA variant (N133S) in a ‘pure’ heteromeric channel formed from a covalently linked WT–mutant tandem dimer, which constrains channel stoichiometry (Supplementary Fig. 2b).

TASK-1 can also co-assemble with TASK-3 subunits to form new heteromeric channels in vivo^25,26^. We therefore co-injected oocytes with WT TASK-3 mRNA plus an equal amount of either WT or mutant TASK-1 mRNA. In this case, all the mutants, including L239P, produced larger heteromeric TASK-1–TASK-3 currents than when WT TASK-1 was used (Fig. 1f). This demonstrates that all these DDSA mutations produce a gain of function in heterozygous TASK-1 and/or heteromeric TASK-1–TASK-3 channels. This effect is clearly different from the loss of function observed for *KCNK3* mutations associated with PAH^27^, including the recently characterized missense variant L214R;^38^ (Supplementary Fig. 2c,d). In further support of our findings, we did not observe any functional effect of a control variant (H141Q) (Supplementary Fig. 2e). This variant, which is also located in the M2 helix, is present in 194 individuals in the gnomAD database^39^ and annotated as likely benign in ClinVar^40^.

### DDSA mutations increase channel open probability

**Fig. 2:**
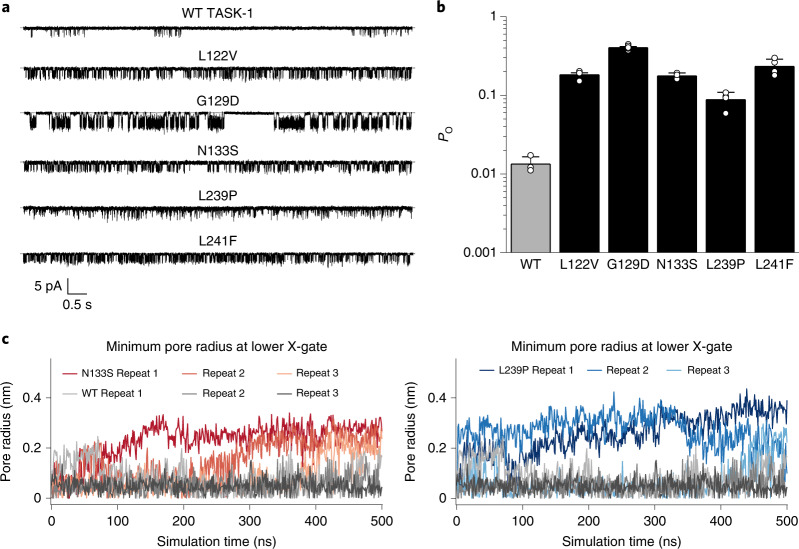
Increased channel open probability due to destabilization of the X-gate. **a**, Representative single-channel recordings of DDSA mutants at a holding potential of −160 mV. **b**, Single-channel *P*_o_ values for each mutant recorded at −160 mV. WT (*n* = 3), N133S (*n* = 3), L239P (*n* = 4), G129D (*n* = 4), L122V (*n* = 4), L241F (*n* = 4) and L122P (*n* = 5). All mutant *P*_o_ values differ from that for WT (*P* < 0.01, two-paired *t*-test). Single-channel conductance measurements for each mutant are also reported in Supplementary Table 1. **c**, Plot of the minimum pore radius at the lower X-gate during three independent repeats of molecular dynamics simulations of the WT TASK-1 structure compared to the N133S and L239P mutant structures. These variants destabilize the closed X-gate structure, allowing the channel to open more frequently.

The whole-cell currents (*I*) described above are a product of the number of channels in the membrane (*N*), their individual open probability (*P*_o_) and single-channel conductance (*γ*). However, trafficking of mutant proteins to the membrane is normally impaired by mutations and rarely increased; additionally, membrane trafficking in an oocyte does not necessarily reflect what happens in more complex native cells, especially neurons^18,41^. We therefore measured the properties of single TASK-1 channels for all of these DDSA variants. Similarly to previous reports^42^, we found that the *P*_o_ of WT TASK-1 was extremely low (*P*_o_ of ~0.02) with a single-channel conductance of ~13 pS. However, homomeric mutant channels for all of the DDSA mutant channels exhibited a markedly increased open probability ranging from 10- to 50-fold greater than that for WT TASK-1, and with the exception of G129D all had a similar single-channel conductance (Fig. 2a,b, Supplementary Fig. 2f,g and Supplementary Table 1). The increased *P*_o_ of the N133S mutant is also consistent with previous reports of other variants at this position in both TASK-3 and TASK-1 (refs. ^24,43^) and indicates that changes in the intrinsic gating properties of these DDSA mutant channels are likely responsible for their increased currents and gain-of-function phenotype.

### DDSA mutations destabilize the closed X-gate in TASK-1

To investigate possible mechanisms by which these DDSA mutations produce an increase in channel activity, we examined the structure of TASK-1. Destabilization of the interactions that hold the X-gate closed will increase the frequency of channel openings (that is, increase *P*_o_) and is consistent with the location of the DDSA variants near this gating motif. In particular, the recurrent N133S variant on M2 involves changes to an amino acid that participates in a hydrogen bond predicted to stabilize the X-gate when closed^24,43^. To investigate this further, we examined multiple variants at this position, all of which resulted in a gain of function, indicating the critical role of this interaction (Supplementary Fig. 3a,b).

In addition, we performed molecular dynamics simulations on WT and both N133S and L239P mutant channel structures. These structures were embedded within a lipid bilayer, and multiple repeats of equilibrium simulations were each run for 500 ns. The results show that the lower X-gate remained firmly closed in all simulations of the WT channel, indicating the stability of this structure. However, the lower gate quickly began to open in simulations of both the N133S and L239P mutant channels, indicating that both these DDSA variants destabilize the closed state structure (Fig. 2c). Interestingly, we previously proposed that a twist and straightening was required to open the X-gate^24^, and similar movements were observed in simulations of the N133S mutant channel (Supplementary Fig. 3c,d).

The location of these variants around the X-gate combined with their ability to destabilize the closed state structure together with their effects on *P*_o_ strongly suggest that they promote opening of this lower gate to produce defective ‘X-gating’. These findings are also consistent with the apparent lack of a dominant filter gate in TASK channels^33,34^. However, some regulation of channel activity can occur via the filter because inhibition by external H^+^ involves titration of a histidine (H98) adjacent to the filter^7,8^. To exclude the possibility of major effects on any such filter-gating mechanism, we measured the external pH sensitivity of the mutant channels (Supplementary Fig. 4a). These results showed that, similarly to WT TASK-1, but unlike H98A pH-sensor mutant channels, all DDSA mutants still exhibited pronounced inhibition by H^+^ at an external pH of 6.0, suggesting that this mechanism remains largely unaffected. However, we cannot exclude the possibility of any additional allosteric effects these mutants may have on the filter itself or any possible coupling between the filter and X-gate.

### DDSA mutations cause dysfunctional GPCR-mediated inhibition

Although the L239P variant results in increased whole-cell currents for heteromeric TASK-1–TASK-3, it produces similarly sized ‘heterozygous’ WT–L239P currents when coexpressed with WT TASK-1 (Fig. 1d–f). This indicates that other defects are also likely to contribute to the pathogenic potential of these DDSA mutations. TASK channels have been shown to be inhibited in vivo by multiple hormones and transmitters, including ATP, thyrotropin-releasing hormone, serotonin, glutamate, catecholamines and acetylcholine, which all act through GPCR signaling via G proteins of the Gα_q/11_ subclass (Gα_q_)^10,14,18,35^. Furthermore, mutations affecting residues in M2 and in the X-gate itself have been shown to impair this process^35,36^. We therefore examined GPCR regulation of the DDSA mutants.

**Fig. 3:**
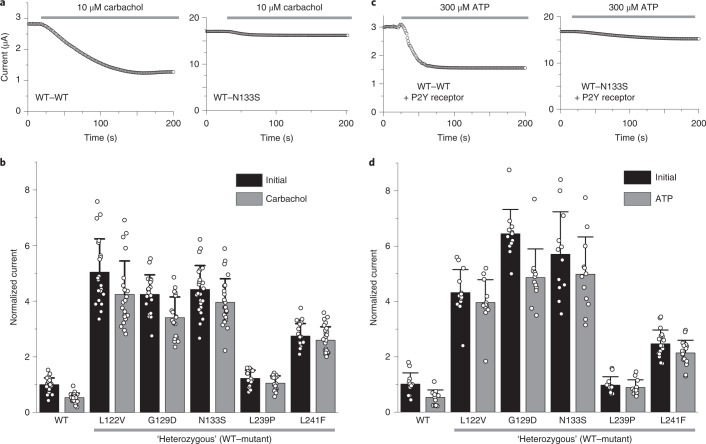
Dysfunctional GPCR-mediated inhibition in DDSA mutants. **a**, Representative currents at +50 mV of WT TASK-1 channels (WT-WT) and ‘heterozygous’ channels from coexpressed WT and N133S subunits, over time while adding 10 µM carbachol. This concentration produces ~50% inhibition of WT TASK-1. **b**, Currents normalized to the initial WT current for WT TASK-1 coexpressed 1:1 with DDSA mutants before and after addition of 10 µM carbachol. WT TASK-1 (*n* = 6), L122V (*n* = 21), G129D (*n* = 19), N133S (*n* = 24), L239P (*n* = 18) and L241F (*n* = 26); data are presented as mean ± s.d. **c**,**d**, Equivalent recordings for WT TASK-1 coexpressed 1:1 with each DDSA mutant as indicated and the P2Y receptor (1:1:4). The current levels shown are before and after addition of 300 µM ATP normalized to the initial WT current. TASK-1 (*n* = 12), L122V (*n* = 13), G129D (*n* = 12), N133S (*n* = 12), L239P (*n* = 12) and L241F (*n* = 18); data are presented as mean ± s.d. The GPCR-mediated inhibition of mutant channel currents is reduced.

To reflect the heterozygous probands, we coexpressed equal amounts of WT and mutant TASK-1 mRNAs and measured their inhibition by either carbachol via endogenous muscarinic receptors or ATP via a coexpressed P2Y receptor^44,45^. We found that GPCR-mediated inhibition by either approach was markedly impaired in all DDSA mutant homomeric channels (Supplementary Fig. 4b), as well as in all ‘heterozygous’ DDSA mutants coexpressed with WT (Fig. 3a–d). By contrast, GPCR regulation of the ‘benign’ H141Q variant appeared unaffected (Supplementary Fig. 4c).

The impaired GPCR-mediated inhibition therefore amplifies the gain-of-function phenotype of these mutants such that, after GPCR activation, their remaining currents become even greater than with WT TASK-1. Notably, this defective inhibition also occurred in the heterozygous WT–L239P channel, whose current became ~2-fold larger than that of WT after receptor stimulation (Fig. 3b,d). This GPCR insensitivity also affected heteromeric TASK-1–TASK-3 channels that contained mutant TASK-1 subunits (Supplementary Fig. 4d).

### Druggability of DDSA mutations

Unlike many other K2P channels that exhibit relatively poor pharmacology^32^, TASK-1 can be inhibited by a range of potent and highly selective small molecules, including a number of clinically relevant drugs^46^. The most potent inhibitor of TASK-1 known to date is BAY1000493, which binds deep within the pore of the channel^24^.

**Fig. 4:**
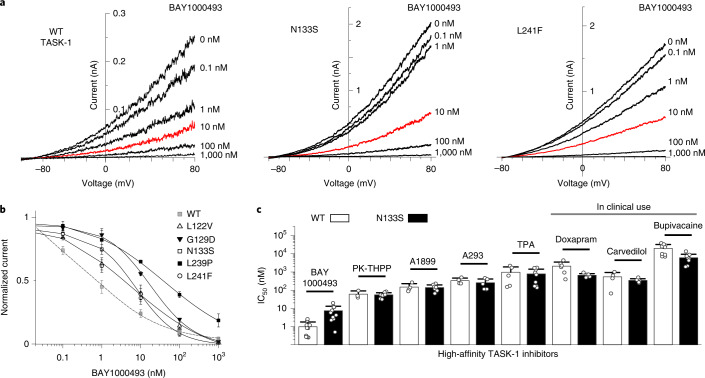
Mutant channel pharmacology. **a**, Representative excised membrane patch recordings of WT TASK-1 and N133S or L241F mutant channel activity in response to different concentrations of BAY1000493 applied to the intracellular side of the patch (10 nM, red). **b**, Corresponding dose–response curves for inhibition of either WT TASK-1 or DDSA mutants by BAY1000493; WT TASK-1 (*n* = 8), L122V (*n* = 10), G129D (*n* = 10), N133S (*n* = 11), L239P (*n* = 3) and L241F (*n* = 10); data are presented as mean ± s.e.m. Values for WT TASK-1 fitted with gray dashed line. **c**, Comparison of IC_50_ values of various high-affinity TASK-1 inhibitors on either WT TASK-1 or the N133S mutant. BAY1000493: WT (*n* = 10), N133S (*n* = 11); PK-THPP: WT (*n* = 3), N133S (*n* = 9); A1899: WT (*n* = 4), N133S (*n* = 9); A239: WT (*n* = 4): N133S (*n* = 7); TPA: WT (*n* = 5), N133S (*n* = 9); doxapram: WT (*n* = 6), N133S (*n* = 6); carvedilol: WT (*n* = 4), N133S (*n* = 7), bupivacaine: WT (*n* = 9), N133S (*n* = 10). Data are presented as mean ± s.d.

Using our ability to record TASK-1 activity in giant excised membrane patches, we measured the inhibition of WT TASK-1 by BAY1000493 (Fig. 4a,b); this produced half-maximal inhibition at a concentration of ≤1 nM (IC_50_ = 970 pM ± 250 pM, *n* = 10), making it the most potent TASK-1 inhibitor known to date. A BAY100493 concentration of 10 nM was very slightly less effective on TASK-1 N133S and L241F mutant channels (Fig. 4a), but full dose–response measurements revealed that all five DDSA mutants are still highly sensitive to this inhibitor within the low nanomolar range, with IC_50_ values ranging from 5 to 23 nM (Fig. 4b). We also examined a range of other known high-affinity TASK channel inhibitors on the N133S mutant; these included PK-THPP, A1899, A293 and tetrapentylammonium (TPA)^46^, all of which we found to have similar IC_50_ values compared to those for WT TASK-1 (Fig. 4c). This mutation also did not affect the reported sensitivity of TASK-1 to inhibition by several drugs already in clinical use, including bupivacaine, carvedilol and the respiratory stimulant doxapram (Fig. 4c).

### Mechanism of GPCR-mediated inhibition

The molecular mechanism of Gα_q_-mediated inhibition of TASK-1 is unclear and has been reported to involve noncanonical effects of pathway activation^44,47^. Normally, Gα_q_ activates phospholipase C (PLC), which hydrolyzes phosphatidylinositol (4,5) bisphosphate (PIP_2_), a lipid that supports the activity of many K2P channels, and so PIP_2_ degradation usually reduces channel activity^10,44,48^. However, it is the concomitant increase in diacylglycerol (DAG) following PIP_2_ hydrolysis that produces TASK channel inhibition, rather than the loss of PIP_2_ itself^44,47–49^. DAG has been shown to directly inhibit TASK-3 channel activity^47^, and we now show that the DAG analog DiC8 also directly inhibits TASK-1 with nanomolar efficacy in excised patches. However, this inhibitory effect is severely impaired by the recurrent DDSA variant, N133S (Supplementary Fig. 4e). PLC activation can also affect the levels of many other signaling lipids, including the endocannabinoid anandamide (AEA), which directly inhibits TASK-1 (ref. ^50^), and we found that the N133S variant also impairs the inhibitory effect of AEA (Supplementary Fig. 4f).

## Discussion

In this study, we describe a new monogenic channelopathy, DDSA, resulting from heterozygous de novo gain-of-function mutations in *KCNK3*. These mutations result in defective X-gating of TASK-1 and increased K^+^ currents through these channels. Although DDSA is rare, this disorder provides a clear example of how the study of a rare genetic disease can illuminate the biology underlying a more common disorder and identify possible therapeutic opportunities for both patients with DDSA and those with sleep apnea.

The expression of TASK-1 in many of the chemosensitive cell types and tissues involved in regulation and mechanical control of breathing means that this channel has long been regarded as an attractive target for the treatment of sleep apnea^51^. However, until now, a clear link between TASK-1 and sleep apnea has not yet formally been established and has even been questioned^52^. Furthermore, the only known genetic mutations affecting TASK-1 were associated with a completely different hypertensive phenotype, PAH^27^.

**Fig. 5:**
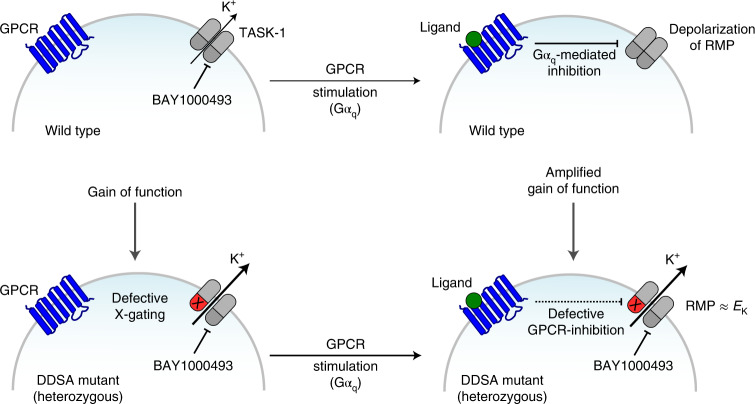
Proposed model for the effect of DDSA mutants on cellular electrical activity. In WT cells, the activity of TASK-1 (that is, homomeric TASK-1 and/or heteromeric TASK-1–TASK-3) channels contributes to the hyperpolarized resting membrane potential (RMP). This activity can be inhibited by Gα_q_-coupled receptor pathways and results in depolarization of the RMP. This gating process involves the cytoplasmic X-gate of TASK-1. However, in cells with a single heterozygous DDSA mutation affecting TASK-1, these variants (marked as X) result in defective closure of the X-gate (marked in red). Consequently, TASK-1 channel activity is increased and/or unresponsive to GPCR-mediated inhibition that amplifies the underlying gain of function. This increased channel activity keeps cells hyperpolarized near the RMP and also uncouples them from regulation by many different GPCR signaling pathways. Notably, mutant channels retain sensitivity to inhibition by several high-affinity small-molecule inhibitors, including BAY1000493. This offers a range of possible therapeutic strategies for these probands and strengthens the rationale for their proposed use in the treatment of sleep apnea.

In addition to sleep apnea, the DDSA probands identified in this study also exhibit a developmental disorder, as well as a number of other cognitive and musculoskeletal phenotypes (Table 1). Other developmental disorders have been linked with sleep disturbances^53^, but just 0.6% of probands within the DDD study database have sleep apnea recorded as one of their phenotypes. Moreover, no causal gene (except *KCNK3*) has sleep apnea recorded in every DDD proband with a diagnostic variant; the universality of sleep apnea in 100% of probands with pathogenic *KCNK3* mutations is therefore highly unusual. It is well known that inappropriate spatiotemporal expression of an ion channel can affect the development of many cell types, especially neurons^54^, and *KCNK3* is expressed at an early stage, especially in the developing CNS^15^. The DDSA phenotype could therefore arise via several possible mechanisms: a general increase in homomeric TASK-1 activity, increased heteromeric TASK-1–TASK-3 activity and/or the resistance of these channels to inhibition by Gα_q_-coupled signaling pathways (Fig. 5). However, the relative contribution of these different molecular mechanisms may be difficult to dissect.

Despite the many phenotypic features shared by the probands, the two with variants in the M4 helix were much less severely affected than those with variants in M2, showing only mild developmental delay and no structural abnormalities combined with only mild or borderline moderate sleep apnea according to their AHI classification, which differs in children compared to adults^55,56^. Interestingly, these M4 variants (L239P and L241F) produced the smallest overall increase in whole-cell currents. This genotype–phenotype correlation might therefore provide some insight into the mechanisms underlying the DDSA phenotypes, but further studies are required to confirm this.

Strikingly, central and/or obstructive sleep apnea was experienced by all (living) probands, severely in the case of those with variants in the M2 helix, and the increased currents and/or lack of GPCR-mediated inhibition producing a gain of function is common to all these variants. The mixture of central and obstructive sleep apnea diagnoses in these probands may reflect the fact that TASK-1 channels are expressed in cells and tissue types likely to affect both respiratory drive and hypoglossal tone. Therefore, defective TASK-1 activity has the potential to manifest in symptoms common to both. We also note that, despite the reported overexpression of *KCNK3* in atrial fibrillation^57^, none of these DDSA probands exhibited any obvious cardiac phenotype.

Our analysis of the functional properties of these mutations provides further insight into the molecular mechanism of TASK-1 gating, in particular, how the X-gate opens and how this is regulated by Gα_q_-coupled pathways. In the structure of TASK-1, the channel pore is occluded by the lower X-gate^24^. The six residues in M4 that form this constriction were previously identified as important for channel regulation by volatile anesthetics and by several Gα_q_-coupled pathways, although the mechanisms involved were unclear^35^.

Our results reveal that the DDSA mutations all cluster near the X-gate in its closed conformation and disrupt the stability of the closed X-gate. In particular, the recurrent N133S variant destabilizes a critical hydrogen bond that holds the X-gate closed and promotes channel opening. Likewise, the inhibitory effect of DAG on TASK-1 channels suggests that it favors stability of the closed state. The fact that all DDSA mutations produce similar effects in both homomeric and heterozygous channels also suggests that disruption of the X-gate within a single subunit is sufficient to open the channel.

The increased whole-cell currents generated by these DDSA mutants result primarily from an increase in channel *P*_o_ rather than an increased number of channels at the cell surface, and in some cases, the mutants may even have decreased surface expression; for example, the L239P mutant has a tenfold increase in *P*_o_ but has similar whole-cell currents to WT TASK-1, an effect that likely results from fewer channels in the membrane. However, Gα_q_-mediated inhibition of the L239P mutant channel is also severely impaired, and so the remaining currents become ~2-fold larger than those for WT TASK-1, therefore still representing a gain-of-function effect.

Fortunately, despite their activatory effects, these mutants still retain sensitivity to a number of small-molecule TASK channel inhibitors, some of which are already in clinical use. This offers a realistic prospect for therapeutic intervention in DDSA probands that may improve their quality of life. Nevertheless, it remains to be determined precisely how TASK-1 dysfunction produces the phenotype observed in DDSA, in particular sleep apnea.

## Methods

### Genetics

Four probands were identified through the United Kingdom-based DDD study, which has been described previously^58,59^. Briefly, probands with severe, undiagnosed developmental disorders and their parents were recruited and phenotyped by referring clinical geneticists in 24 regional genetics services across the National Health Service in the United Kingdom and Ireland. Saliva and/or blood-extracted DNA samples were analyzed at the Wellcome Sanger Institute using massively parallel whole-exome sequencing of the family trio using SureSelect RNA baits (Agilent Human All-Exon V3 Plus with custom ELID C0338371 and Agilent Human All-Exon V5 Plus with custom ELID C0338371). Enriched libraries were analyzed by 75-base paired-end sequencing (Illumina, HiSeq); reads were mapped to GRCh37/UCSC hg19 and variants were called using the Genome Analysis Toolkit (GATK)^60^ and annotated using Ensembl Variant Effect Predictor^61^. DeNovoGear was used to predict likely de novo mutations^62^, and variants were identified for clinical feedback as previously described^63^.

Three probands were identified by the United States-based diagnostics company GeneDx. Using genomic DNA from the proband and parents, the exonic regions and flanking splice junctions of the genome were captured using the Clinical Research Exome kit (Agilent Technologies) or the IDT xGen Exome Research Panel v1.0. Massively parallel (NextGen) sequencing was done on an Illumina system with 100-bp or greater paired-end reads. Reads were aligned to human genome build GRCh37/hg19 and analyzed for sequence variants. Additional sequencing technology and the variant interpretation protocol have been previously described^64^. The general assertion criteria for variant classification are publicly available on the GeneDx ClinVar page (http://www.ncbi.nlm.nih.gov/clinvar/submitters/26957/).

One proband was identified by the Mayo Medical Laboratories in the United States. Whole-exome sequencing was performed on genomic DNA extracted from all samples submitted. The exome was captured using a custom reagent developed by Mayo Clinic and Agilent Technologies. Sequencing was performed on an Illumina HiSeq 2500 Next Generation sequencing instrument, using HapMap sample NA12878 as an internal control. Paired-end 101-bp reads were aligned to a modified human reference genome (GRCh37/hg19) using Novoalign. Sequencing quality was evaluated using FastQC. All germline variants were jointly called through GATK Haplotype Caller and GenotypeGVCF, and each variant was annotated using the BioR Toolkit. Data were filtered and analyzed to identify clinically relevant sequence variants. Variants of interest were confirmed by automated Sanger sequencing.

One proband was identified at the German Institute of Clinical Genetics, Dresden, Germany. DNA was extracted from blood-derived lymphocytes using the QIAamp DNA Blood Mini Kit (Qiagen). Patient as well as parental samples were studied in parallel (trio exome strategy). Exome capture was performed using the IDT xGene Exome Research Panel. Paired-end sequencing (150 nucleotide) was performed with a median target coverage of at least 50-fold on Illumina NextSeq500 sequencing systems. Alignment (mapping to GRCh37/hg19), and variant identification (single-nucleotide variants and indels), annotation and filtering were performed using the CLC Biomedical Genomics Workbench (Qiagen), as described previously^65^. Variants were filtered with a focus on protein-altering variants (missense, frameshift, splice site and premature stop codon) and rarity in public databases (gnomAD, allele frequency below 1% and not more than five homozygous or hemizygous individuals). Additionally, variants were prioritized based on assumed inheritance patterns.

For all studies, candidate de novo mutations in *KCNK3* were visually inspected using the Integrative Genomics Viewer (IGV)^66^. Likely diagnoses were communicated to referring clinical teams for diagnostic validation (including confirmation by Sanger sequencing where appropriate) and discussion with the family.

### Diagnosing sleep apnea

All living probands with pathogenic *KCNK3* mutations were evaluated in a clinical sleep laboratory, where standard diagnostic procedures were followed. AHI and O_2_/CO_2_ saturation statistics in supine/non-supine positions are included in Table 1 (excluding the severest case for whom this was not possible and the mildest case where AHI values were measured after treatment with BiPAP). All available sleep reports and polysomnography data obtained before treatment are reproduced in Supplementary Data 1.

### Electrophysiology

The WT human TASK-1 gene (*KCNK3*) was subcloned into a plasmid vector (pFAW) suitable for in vitro transcription and expression in *Xenopus laevis* oocytes^67^. Mutations were introduced by site-directed mutagenesis and confirmed by sequencing. Unless otherwise stated, a volume of ~18 nl of mRNA was injected into oocytes at a concentration of 110 ng μl^−1^ for either WT protein or mutants (that is, 2 ng of RNA per oocyte). TEVC recordings were performed as previously described^67^. Briefly, after injection of mRNA, oocytes were incubated for 22–24 h at 17.5 °C and measured in ND96 buffer at pH 7.4 (96 mM NaCl, 2 mM KCl, 2 mM MgCl_2_, 1.8 mM CaCl_2_ and 5 mM HEPES). Unless otherwise stated, currents were recorded using a 400-ms voltage step protocol from a holding potential of −80 mV delivered in 10-mV increments between −120 mV and +50 mV and 800-ms ramp protocols from −120 to +50 mV. All recorded traces were analyzed using Clampfit (Axon Instruments), and graphs were plotted using Origin2019b (OriginLab Corporation). Unless otherwise described, all results shown are reported as mean ± s.d. and were obtained with oocytes from at least three independent batches with more than 23 recordings per construct for comparison of current levels and at least 12 recordings for GPCR-mediated inhibition studies. Where relevant, significance values are reported in the figure legends with associated *P* values. Currents of DDSA mutants were compared separately to WT currents in two-sample *t*-tests under conditions of normality for data distribution and equal variance. Normality at a 0.05 level was tested using a Shapiro–Wilk test.

Single-channel currents in cell-attached patches from *Xenopus* oocytes were recorded in symmetrical 140 mM KCl solutions containing 10 mM HEPES (pH 7.4 with KOH). Data were filtered at 2 or 5 kHz and recorded at a 200-kHz sampling rate with the program Clampex on an Axopatch 200B amplifier. Data analysis was performed using Clampfit. Channel *P*_o_ was determined from single-channel recordings with a duration of 1–4 min, which exhibited only one main open level; each single channel was recorded at four different membrane potentials (Supplementary Fig. 2g). For WT TASK-1 channels, *P*_o_ was estimated from recordings that exhibited only one main open level and in which the number of detected single openings was >3,000, giving a confidence value *P* > 0.999 for having a single channel^68^. Comparison of *P*_o_ values was done using a two-sample Student’s *t*-test with assumptions of normality and equal variance based upon the length of the recordings^69^. Single-channel current amplitude of the L239P mutant channels was estimated as follows: first, a closed level histogram for current values above 0 pA was approximated as a mirror image of the values below 0 pA and the resulting histogram was subtracted from the data. The mean single-channel current amplitude *i* was then calculated from the following equation where *N*_*k*_ represents the number of openings with a current value *i*_*K*_:

$$i = \mathop {\sum }\limits_k \frac{{i_kN_k}}{{N_k}}$$

Giant excised membrane patch measurements in inside-out configuration under voltage-clamp conditions were made at room temperature 72–120 h after injection of 50 nl of channel-specific mRNA into *X. laevis* oocytes. Thick-walled borosilicate glass pipettes had resistances of 0.25–0.35 MΩ (tip diameter of ~15–30 µm) and were back-filled with extracellular solution containing 4 mM KCl, 116 mM NMDG, 10 mM HEPES and 3.6 mM CaCl_2_ (pH was adjusted to 7.4 with KOH/HCl). Bath solution was applied to the cytoplasmic side of the excised giant patches via a gravity flow multibarrel application system and had the following composition: 120 mM KCl, 10 mM HEPES, 2 mM EGTA and 1 mM pyrophosphate. Currents were acquired with an EPC10 USB amplifier and HEKA PatchMaster 2×91 software. The sampling rate was 10 kHz, and the analog filter was set to 3 kHz (−3 dB). Voltage ramp pulses (−80 mV to +80 mV) were applied from a holding potential (*V*_H_) of −80 mV for 0.8 s with an interpulse interval of 9 s and were analyzed at a given voltage of +40 mV. The relative steady-state levels of inhibition for the indicated blocker were fitted with the following Hill equation:

$$Y = \frac{{{\textrm{base}} + ({\textrm{max}} - {\textrm{base}})}}{{\left( {\frac{{X_{{\textrm{half}}}}}{X}} \right)^H}}$$

where base is the inhibited (zero) current, max is the maximum current, *x* is the blocker concentration, *x*_half_ is the value of concentration for half-maximal occupancy of the blocker binding site and *H* is the Hill coefficient.

TPenA-HCl, bupivacaine hydrochloride monohydrate, A293 (AVE1231), doxapram hydrochloride and 1,2-dioctanoyl-*sn*-glycerol (DiC8) were purchased from Sigma-Aldrich. PK-THPP, A1899, carvedilol and anandamide (AEA) were purchased from Tocris Bioscience. The TASK-1 K2P-specific inhibitor BAY1000493 was provided by Bayer AG. All compounds were stored as stock solutions (10–100 mM) at −80 °C and were diluted in intracellular bath solution to final concentrations before each measurement.

### Molecular dynamics

Simulations were done in GROMACS 2018 using the CHARMM36 force field. Missing atoms in the protein structure (PDB: 6RV2) were repaired using SWISS-MODEL, with mutants generated in PyMOL. Each structure was embedded into phosphatidylcholine (POPC) membranes in independent triplicates and solvated by aqueous NaCl at 150 mM. Temperature and pressure were maintained at 310 K and 1 bar using the velocity-rescaling thermostat and a semi-isotropic Parrinello and Rahman barostat, with coupling constants of 0.1 ps and 1 ps, respectively. Long-range electrostatic interactions were treated using the particle mesh Ewald method. The integration time step was 2 fs. Covalent bonds were constrained through the LINCS algorithm. Pore radii along the channel pathway were determined using the CHAP program^70^.

### Reporting summary

Further information on research design is available in the Nature Research Reporting Summary linked to this article.

## Online content

Any methods, additional references, Nature Research reporting summaries, source data, extended data, supplementary information, acknowledgements and peer review information; details of author contributions and competing interests; and statements of data and code availability are available at 10.1038/s41588-022-01185-x.

## Supplementary information

Supplementary Information Supplementary Figs. 1–4 and Supplementary Table 1.

Reporting Summary

Supplementary Data 1 Supplementary data for sleep phenotype.

## Data Availability

The authors confirm that all relevant data are included in the article and/or its Supplementary Information. For some probands, only AHI and nadir SpO_2_ values from their original diagnoses are available. The available sleep reports are reproduced in their original form in Supplementary Data 1. Where relevant, individual data points for the functional measurements of channel activity are shown in each figure. Structural data for TASK-1 were obtained from the PDB under accession number 6RV2.

## Code availability

All software used in the study are publicly available as described in the Methods and Reporting Summary.
